# Synthesis and Leishmanicidal Activity of 1-[5-(5-Nitrofuran-2-yl)-1, 3, 4-Thiadiazole-2-yl]-4-BenzoylePiperazines

**Published:** 2017

**Authors:** Alireza Foroumadi, Hadi Adibi, Sussan Kabudanian Ardestani, Samira Shirooie, Arezoo Bozorgomid, Ali Jafari

**Affiliations:** a *Department of Medicinal Chemistry, Faculty of Pharmacy and Pharmaceutical Sciences Research Center, Tehran University of Medical Sciences, Tehran, Iran.*; b *Pharmaceutical Sciences Research Center, Faculty of Pharmacy, Kermanshah University of Medical Sciences, Kermanshah, Iran. *; c *Department of Biochemistry, Institute of Biochemistry and Biophysics, University of Tehran, Tehran, Iran.*; d *Student’s Research Committee, Kermanshah University of Medical Sciences, Kermanshah, Iran.*; e *Department of Medical Parasitology and Mycology, School of Public Health, Tehran University of Medical Sciences, Tehran, Iran.*

**Keywords:** 1, 3, 4-Thiadiazole, Nitrofuran, Antileishmanial activity, Leishmania Major, Promastigote

## Abstract

A series of **(**5-nitrofuran-2-yl)-1, 3, 4-thiadiazole-2-yl derivatives 6a–6e have been synthesized and screened for *in-vitro* anti-leishmanial activity against the promastigote form of *L. major*. The structure of Schiff bases were confirmed by ^1^H NMR, IR. Screening results indicate that all of the designed and synthesized final compounds (6a-6e) significantly reduced the viability of promastigotes of *L. major* in comparison toglucantime (IC_50_ 3× 10^3^ μg/mL). Meta and Para substitutions in benzene ring containing compounds were more potent than other derivative and the most potent compounds were 6d, 6e with IC_50 _value 94 µm and 77.6 µm, respectively. The experimental data proposes that **(**5-nitrofuran-2-yl)-1, 3, 4-thiadiazole-2-yl derivatives may be further investigated as a candidate drug for treatment of cutaneous leishmaniasis.

## Introduction

Leishmaniasis is an infection caused by the protozoan parasites belonging to the genus *Leishmania* and transmitted by the bite of an infected *Phlebotomus* in the Old World and *Lutzomyia* in the New World (1). 

This disease is still one of the world’s most neglected diseases, because is present in 98 countries some of which are among the poorest in the world. According to WHO data, 350 million people in 88 countries are considered at risk of contracting leishmaniasis, and some 2 million new cases occur annually (2). More than 90% of the CL cases occur in six countries, Iran, Afghanistan, Syria, Saudi Arabia, Brazil, and Peru (3, 4). In Iran, almost 20,000 people are infected with cutaneous leishmaniasis each year (5). 

The present control measures depends on chemotherapy including pentavalent antimonial, miltefosine, paromomycin and amphotericin B as the standard drugs for treatment but these drugs are expensive and long-term administration (6). The drug resistance, severe toxic side effects to this of drugs has been reported from many parts of the world (6, 7). Therefore there is an urgent need for an effective treatment for leishmaniasis that is safe, inexpensive, and orally available.

**Scheme 1:**
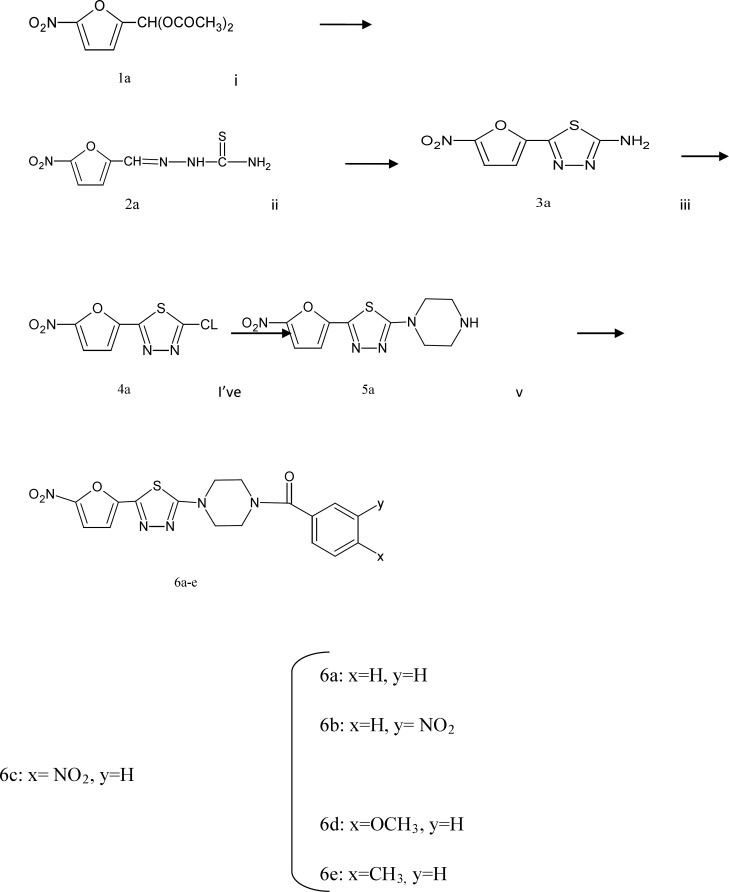
Reagents and conditions: (i) thiosemicarbazide, EtOH, HCl, reflux, 1 h. (ii) NH_4_Fe(SO4)_2_.12H_2_O, H_2_O, reflux, 16 h. (iii) NaNO_2_, HCl, Cu, 0 C! rt, 3 h. (iv) piperazine, EtOH, reflux, 1 h (v) benzoyl chloride, 3-nitro benzoyl chloride, 4-nitro benzoyl chloride, 4-methoxy benzoyl chloride, 4-methyl benzoyl chloride, pyridine, EtOH, rt , 24 h

**Table 1:** Effect of synthesized compounds on the growth of *Leishmania major*promastigotes

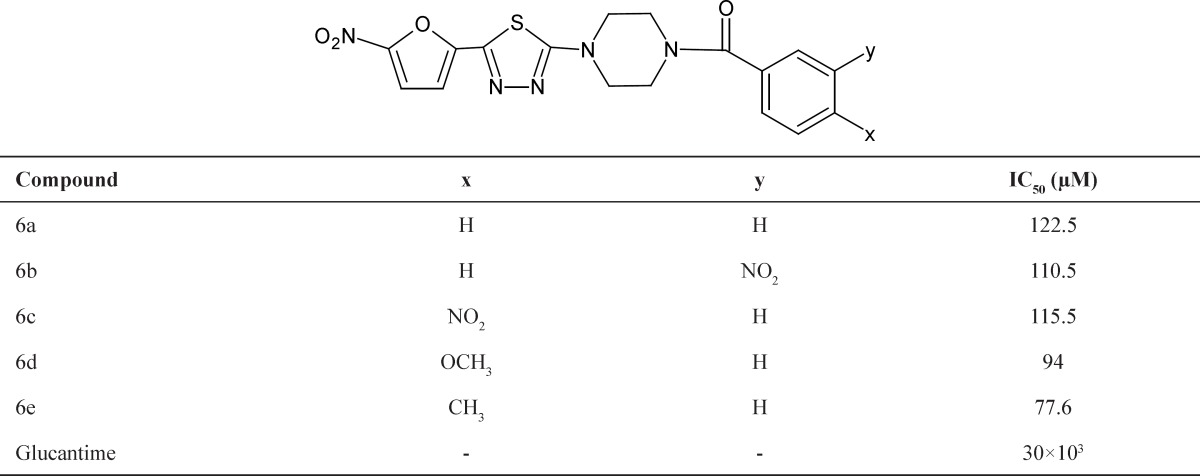

In the last decades, Heterocyclic ring systems ﬁnd great importance in the medicinal chemistry research due to their interesting biological and synthetic applications. Among different heterocyclic compounds 1, 3, 4-thiadiazoles and their derivativeshave gained importance. Thiadiazole is a 5-membered ring system containing hydrogen-binding domain, sulfur atom, and two-electron donor nitrogen system that exhibit broad spectrum of pharmacological properties such as: antibacterial (8-10), Antifungal (11), Anti-tubercular (12), Antiviral (13), Antioxidant (14), Antitumoral (10,15), Anti-inﬂammatory (9) and etc.It is known that 1, 3, 4- thiadiazole ring are included in the structure of various drugs like Acetazolamide (16). Sulfaethidole, Sulfamethizole, Cefazodone, Cefazoline (17). Literature surveys demonstrated the promising anti-leishmaniasis activity of 1, 3, 4-thiadiazoles derivatives (18-20). Considering the biological signiﬁcance of 1, 3, 4- thiadiazoles, we decided to synthesize some novel 1-[5-(5-nitrofuran-2-yl)-1, 3, 4-thiadiazole-2-yl]-4-benzoylepiperazines (6a-e) derivatives and screen them for antileishmaniasis activity.

*Chemistry*

All chemical and solvent used in this study were purchased from Merckand used without further puriﬁcation. The melting points were determined on an electrothermal digital melting point apparatus. Infrared (IR) spectra were recorded using a Shimadzu 470 spectrophotometer (potassium bromidedisks). Proton nuclear magnetic resonance (^1^H-NMR) spectra was recorded using a Bruker500 MHz spectrometer and chemical shifts are reported in parts per million (ppm) relative to tetramethylsilane as internal standard. 

*Synthesis of the compounds*

*Chemistry*

The synthetic pathway to target compounds 6a-e is shown in Scheme 1. The 2-amino-5-(5-nitro-2-furyl)-1, 3, 4-thiadiazole 3a was obtained by oxidative cyclization of 5-nitrofurancarboxaldehyde thiosemicarbazone 2a. Diazotization of 3a in hydrochloric acid in the presence of copper powder gave 2-chloro-5-(5-nitro-2-furyl)-1, 3, 4-thiadiazole (4a).

Reaction of compound 4a with piperazine in refluxing ethanol gave compound 5a.

The reaction of 5a with benzoyl chloride yielded compound 6a.

Similarly, the reaction of compound 5a with 3-nitro benzoyl chloride, 4-nitro benzoyl chloride, 4-methoxy benzoyl chloride, 4-methyl benzoyl chloride gave the corresponding compounds 6b-e, respectively (16).

## Experimental

*Chemistry*

Chemical reagents and all solvents in this study were purchased from Merck AG Chemical and used without further puriﬁcation.

Melting points were determined on a Kofler hot stage apparatus and at room temperature. The IR spectra were obtained on a Shimadzu 470 spectrophotometer (potassium bromide dicks).

¹H NMR spectra were recorded on a Varian unity 80 spectrometer and chemical shifts are reported in parts per million (d) relative to tetramethylsilane (TMS) as an internal standard. Merck silica gel 60 F254 plates were used for analytical TLC.

4.1.1.1-benzoyl-4-[5-(5-nitro-2-furyl)-1, 3, 4-thiadiazol-2-yl] piperazine (6a).Yeild 74%; mp 220-222 °C; IR (KBr) νmax: 1380 & 1510 (stretch NO_2_), 1690 (stretch C=O), 3000-3200 (stretch CH, sp² aromatic), 1480 & 1500 (stretch C=C, aromatic), 2915cm‾¹ (stretch CH, sp³ aliphatic).

¹H-NMR (DMSO, 200MHZ); δ (ppm): 7.94 (d, j=6, 2H, H_1_, H_6_- phenyl), 7.64 (t*, j*=6, 2H, H_3, _H_5- phenyl_), 7.51 (m, *j=*6, 3H, H_3_, H_4-_ furan & H_4- _phenyl), 3.84 -3.40 (m, 8H- piperazine).

4.1.2.1-(3-nitro benzoyl-4-[5-(5-nitro-2-furyl)-1, 3,4-thiadiazol-2-yl] piperazine (6b).Yeild 96%; mp 198-200 °C; IR (KBr) νmax: 1350 & 1510 (stretch NO_2_), 1700 (stretch C=O), 3020-3200 (stretch CH, aromatic), 1440 & 1630 (stretch C=C, aromatic), 2950cm‾¹ (stretch CH, sp³ aliphatic).sp²

¹H-NMR (DMSO, 200 MHZ); δ (ppm): 8.58 (s, 1H,H_2_- phenyl), 8.42 (d, *j*=6, 1H, H_4_- phenyl), 8.33 (d, *j*=8, 1H, H_6- _phenyl), 7.78 (t ,*j*=8, 1H, H_5-_ phenyl), 7.39 (t, *j*=4, 2H, H_3,_, H_4-_ furan), 3.80- 3.01 (m, 8H- piperazine). 

4.1.3. 1-(4-nitro benzoyl-4-[5-(5-nitro-2-furyl)-1, 3, 4-thiadiazol-2-yl] piperazine (6c).Yeild 80%; mp 213-215 °C; IR (KBr) νmax: 1350 & 1500 (stretch NO_2_), 1680 (stretch C=O), 3000-3200 (stretch CH, aromatic), 1440 & 1610 (stretch C=C, aromatic), 2900cm‾¹ (stretch CH, sp³ aliphatic).

¹H-NMR (DMSO, 200 MHZ); δ (ppm): 8.57 (d,* j*=6, 2H, H_3_, H_4_- furan), 8.31 (d, *j*=8, 2H, H_2_, H_6_- phenyl), 8.16 (d,* j*=8, 2H, H_3_, H_5_- phenyl), 3.75 (m, 8H- piperazine).

4.1.4. 1-(4-methoxy benzoyl-4-[5-(5-nitro-2-furyl)-1, 3,4-thiadiazol-2-yl] piperazine (6d).Yeild 74%; mp 212-214 °C; IR (KBr) νmax: 1350 & 1530 (stretch NO_2_), 1690 (stretch C=O), 3000-3200 (stretch CH, sp² aromatic), 1430 & 1600 (stretch C=C, aromatic), 2900 (stretch CH, sp³ aliphatic), 1160 & 1250cm‾¹ (stretch C-O-methoxy).

¹H-NMR (DMSO, 200 MHZ); δ (ppm): 7.89 (d, *j*=7, 2H, H_2_, H_6_- phenyl), 7.44 (d, *j*=2, 2H, H_3_, H_4_- furan), 6.98 (d,* j*=7, 2H, H_3_, H_5_- phenyl), 3.81 (s, 3H- methoxy), 3.66- 3.36 (m, 8H- piperazine). 

4.1.5. 1-(4-methyl benzoyl-4-[5-(5-nitro-2-furyl)-1, 3, 4-thiadiazol-2-yl] piperazine (6e).Yeild 47%; mp 220-222 °C; IR (KBr) νmax: 1360 & 1520 (stretch NO_2_), 1690 (stretch =O), 3050-3200 (stretch CH, sp² aromatic), 1440 & 1610 (stretch C=C, aromatic), 2900cm‾¹ (stretch CH, sp³ aliphatic).

¹H-NMR (DMSO, 200 MHZ); δ (ppm): 7.79 (d,* j*=8, 2H, H_2_, H_6_- phenyl), 7.40 (d, *j*=4, 2H, H_3_, H_4_-furan), 7.21 (d, *j*=8, 2H, H_3_, H_5_- phenyl), 3.32 (m, 8H- piperazine), 2.31 (s, 3H- methyl).

*Parasite culture*

The *L. major* strain MRHO/IR/75/ER was provided from Pasteur institute, Tehran (Iran). Promastigotes of*L. major* were cultured *in-vitro* at 26ºC in RPMI 1640 complete medium containing 10% FBS, 4 mm L-glutamine, 25 mm HEPES, 0.1 mm non-essential amino acid, 1 mm sodium pyruvate, 50 µm 2-ME, streptomycin (100 µg/mL), Penicillin (100 u/mL). 

The parasites were collected from the logarithmic phase.

*In-vitro evaluation of anti-promastigote activity*

To determine the 50% inhibitory concentration (IC_50_) against *L.major*, The synthesized compounds were dissolved in dimethyl sulphoxide (DMSO) ata concentration of 0.01% and diluted with RPMI medium. Promastigotes were counted in a Neubauerhemocytometer and seeded at 2.6× 10^6^cells per well in 96-well plastic plates containing different concentrations of the compounds and RPMI 1640 complete medium, with a ﬁnal volume of 200 µL. Cultured cells in the presence of DMSO were used as viability controls, while glucantime were used as Leishmanicidal controls. After 24 h. incubation in 25°C, parasite viability was determined using the MTT assay (3-[4, 5-dimethylthiazol-2-yl]-2, 5-diphenyltetrazolium bromide; thiazolyl-blue, Sigma, Germany).Assays were performed twice with three replicates per each concentration tested.

*Statistical analysis*

The results were deﬁned as the mean values of at least three experiments. Statistical analysis was carried out by using the SPSS ver. 16 software.

## Results and Discussion

For evaluation of anti-leishmanial properties of target compounds, the *in-vitro* activity was assessed against promastigote (log phase) forms of *L. major*. The inhibitory effects of compounds against promastigotes forms of *L. major* were measured after 72 h. following established procedures, at concentrations ranging from 25 to 100 μm. The IC_50_ values registered after 24 h. of exposure are represented in Table 1 including Glucantime as the reference drug.

Our selection to synthesis and anti-leishmanial activity evaluation 1-[5-(5-nitrofuran-2-yl)-1, 3, 4-thiadiazole-2-yl]-4-benzoylepiperazines, in this paper was based on reports of the literature indicating that 5-(5-nitrofuran- 2-yl)-1, 3, 4-thiadiazoles moiety has been effective against promastigote and amastigote forms *L. major* strain *in-vivo* and *in-vitro *(19, 20)

In the present work, all of the synthesized compounds (6a-e) were more effective than the reference drug. The activity is increased in the following order: 6e > 6d > 6b > 6c > 6a. Compound 6e was found to be the most active compound in this series (IC_50_ = 77.6 μm). (Screening results indicate that Meta and Para substitutions in benzene ring increase the efficacy and decrease IC_50_ of the synthesis compounds and compounds with electron donor groups (as in compounds 6e, 6d) have greater activity than the others.

## Conclusion

In summary, a series of 1-[5-(5-nitrofuran-2-yl)-1,3,4-thiadiazole-2-yl]-4-benzoylepiperazines were synthesized and evaluated for their *in-vitro* inhibitory activity against the *Leishmania* parasite. All of the target compounds exhibited good anti-leishmanial activity against the promastigote form of *L. major*. These data encourage furthermore studies evaluating the effect on intracellular amastigotes *in-vitro*, *in-vivo* efficacy and cytotoxicity of the compounds. 

## References

[1] Nagill R, Kaur S (2011). Vaccine candidates for leishmaniasis: a review. Int. Immunopharmacol.

[2] Ashford R, Desjeux P (1992). Estimation of population at risk of infection and number of cases of leishmaniasis. Parasitol. Today.

[3] Ashford R, Desjeux P (1992). Estimation of population at risk of infection and number of cases of leishmaniasis. Parasitol. Today.

[4] Hamzavi Y, Khademi N (2015). Trend of Cutaneous Leishmaniasis in Kermanshah Province, West of Iran from 1990 To 2012. Iran. J. Parasitol..

[5] Mohebali M (2013). Visceral leishmaniasis in Iran: review of the epidemiological and clinical features. Iran. J. Parasitol..

[6] Kedzierski L, Zhu Y, Handman E (2006). Leishmania vaccines: progress and problems. Parasitology.

[7] Hadighi R, Mohebali M, Boucher P, Hajjaran H, Khamesipour A, Ouellette M (2006). Unresponsiveness to Glucantime treatment in Iranian cutaneous leishmaniasis due to drug-resistant Leishmania tropica parasites. PLoS. Med..

[8] Abdel-Wahab BF, Abdel-Aziz HA, Ahmed EM (2009). Synthesis and antimicrobial evaluation of some 1, 3-thiazole, 1, 3, 4-thiadiazole, 1, 2, 4-triazole, and 1, 2, 4-triazolo [3, 4-b][1, 3, 4]-thiadiazine derivatives including a 5-(benzofuran-2-yl)-1-phenylpyrazole moiety. Monatsh. Chem..

[9] Kadi AA, Al-Abdullah ES, Shehata IA, Habib EE, Ibrahim TM, El-Emam AA (2010). Synthesis, antimicrobial and anti-inflammatory activities of novel 5-(1-adamantyl)-1, 3, 4-thiadiazole derivatives. Eur. J. Med. Chem..

[10] Foroumadi A, Sorkhi M, Moshafi MH, Safavi M, Rineh A, Siavoshi F, Shafiee A, Emami S (2009). 2-Substituted-5-nitroheterocycles: In-vitro anti-Helicobacter pylori activity and structure-activity relationship study. Med. Chem..

[11] Matysiak J, Malinski Z (2007). 2-(2, 4-dihydroxyphenyl)-1, 3, 4-thiadiazole analogues: Antifungal activity in- vitro against Candida species. Russ. J. Bioorg. Chem..

[12] Foroumadi A, Asadipour A, Mirzaei M, Karimi J, Emami S (2002). Antituberculosis agents V Synthesis, evaluation of in-vitro antituberculosis activity and cytotoxicity of some 2-(5-nitro-2-furyl)-1, 3, 4-thiadiazole derivatives. Il Farmaco..

[13] Invidiata F, Simoni D, Scintu F, Pinna N (1996). 3, 6-Disubstituted 1, 2, 4-triazolo [3, 4-b][1, 3, 4] thiadiazoles: synthesis, antimicrobial and antiviral activity. II Farmaco (Societa chimica italiana: 1989).

[14] (Khan I, Ali S, Hameed S, Rama NH, Hussain MT, Wadood A, Uddin R, Ul-Haq Z, Khan A, Ali S, Choudhary MI (2010). Synthesis, antioxidant activities and urease inhibition of some new 1, 2, 4-triazole and 1, 3, 4-thiadiazole derivatives. Eur. J. Med. Chem..

[15] Wei M-X, Feng L, Li X-Q, Zhou X-Z, Shao ZH (2009). Synthesis of new chiral 2, 5-disubstituted 1, 3, 4-thiadiazoles possessing γ-butenolide moiety and preliminary evaluation of in-vitro anticancer activity. Eur J. Med. Chem..

[16] Tsuji K, Ishikawa H (1994). Synthesis and anti-pseudomonal activity of new 2-isocephems with a dihydroxypyridone moiety at C-7. Bioorg. Med. Chem. Lett..

[17] Ferrer S, Borrás J, Miratvilles C, Fuertes A (1989). Coordination behavior of acetazolamide (5-acetamido-1, 3, 4-thiadiazole-2-sulfonamide) F synthesis, crystal structure, and properties of bis (acetazolamidato) tetraamminenickel (II). Inorg. Chem..

[18] Behrouzi-Fardmoghadam M, Poorrajab F, Ardestani SK, Emami S, Shafiee A, Foroumadi A (2008). Synthesis and in-vitro anti-leishmanial activity of 1-[5-(5-nitrofuran-2-yl)-1, 3, 4-thiadiazol-2-yl]-and 1-[5-(5-nitrothiophen-2-yl)-1, 3, 4-thiadiazol-2-yl]-4-aroylpiperazines. Bioorg. Med. Chem..

[19] Poorrajab F, Ardestani SK, Foroumadi A, Emami S, Kariminia A, Behrouzi-Fardmoghadam M, Shafiee A (2009). Selective leishmanicidal effect of 1, 3, 4-thiadiazole derivatives and possible mechanism of action against Leishmania species. Exp. Parasitol..

[20] Tahghighi A, Marznaki FR, Kobarfard F, Dastmalchi S, Mojarrad JS, Razmi S, Ardestani SK, Emami S, Shafiee A, Foroumadi A (2011). Synthesis and antileishmanial activity of novel 5-(5-nitrofuran-2-y1)-1, 3, 4-thiadiazoles with piperazinyl-linked benzamidine substituents. Eur. J. Med. Chem..

